# On Modern Progress in Ophthalmic Medicine and Surgery

**Published:** 1893-12-09

**Authors:** Robert Brudenell Carter

**Affiliations:** Consulting Ophthalmic Surgeon to St. George's Hospital


### On Modern Progress in Ophthalmic Medicine and Surgery.

By Robert Brudenell Carter, F.R.C.S., Consulting Ophthalmic Surgeon to St. George's Hospital.

From the first introduction of the ophthalmoscope into practice, it began daily to afford increased knowledge with regard to the internal diseases of the eye, enabling surgeons to discriminate between maladies which had previously been confounded together, and bringing to light many conditions, the existence of which had not previously been suspected. Of all its early triumphs, however, the greatest was the discovery of the essential nature of glaucoma, and of the means by which the primary stages of this condition could be prevented from passing into blindness.

Prior to the period of which I am now writing, glaucoma had long been known as a malady which was preceded or ushered in by certain well defined symptoms, such as the appearance of haloes around candle flames, a rapid increase of presbyopia, the occurrence of temporary obscurations of vision, and progressive narrowing of the field, especially on the nasal side. It was known that these symptoms were usually followed, sooner or later, by attacks of severe pain, and that they terminated in total blindness, accompanied by stony hardness of the eyeball; but this hardness was regarded as a result of intercurrent internal inflammation, and not as an essential part of the morbid process. The pains of glaucoma were often attributed to "rheumatism" or to "gout"; and the disease as a whole may be found described, in text-books published during the first half of the century, as "acute internal arthritic ophthalmia."

When the ophthalmoscope was applied to the examination of eyes in which the symptoms of impending glaucoma had declared themselves, the first consequent discovery was that the surface of the optic nerve, instead of being slightly prominent, as in the healthy state, was depressed; sometimes to such an extent as to constitute a definite cup or excavation, the floor of which was formed by the surface of the nerve itself, and the boundaries by the edges of the scleral foramen, through which the nerve fibres find entrance into the eye. It became evident, when once these facts had been established by a sufficient number of observations, that the optic nerve surface, as the place of least resistance, was the first to yield to pressure exerted from within the eye; that the increased tension or hardness of the advanced stages, instead of being an accidental complication, was a primary and essential factor of the disease; and that glaucoma was nothing more than distension of the eye from hypersecretion of fluid, or from the inadequate absorption or removal of a secretion of the ordinary amount; while the liability to attacks of pain and inflammation, or, in other words, the acute, sub-acute, or chronic character of the case, was simply a measure of the degree of rapidity with which the amount of fluid had become excessive. As soon as these conclusions had been reached, they afforded definite indications of the direction in which a cure might be sought, and they induced surgeons to consider by what means, if any, intra-ocular tension might be diminished.

The first practical suggestion for this purpose came from Albrecht von Graefe, who had observed that the removal of a piece of iris, in order to make an artificial lateral pupil in cases of central opacity of the cornea, rendered the eye permanently softer than it had been before the operation. He determined to perform an experimental iridectomy on any cases of glaucoma which came under his treatment; and, so early as in 1857, he was able to publish cases which more than justified his most sanguine expectations. They showed that glaucoma had been removed from the category of incurable diseases, and that a great triumph had been won for surgery. The operation had been suggested by purely empirical considerations, and for many years its reputation rested upon practical experience alone, no adequate explanation having been given of the manner in which its effects were produced. It was conjectured that more room was afforded within the eye by the removal of a portion of its contents; that the aqueous humour was secreted, partly at least, by the posterior surface of the iris, so that the removal of a portion of this membrane would diminish the amount or the rapidity of the secretion; and, finally, that the cicatrix of the external incision was more permeable to fluid, and permitted a more free exudation, than was permitted by the corneo-scleral junction in its natural state. Speculations of this kind were of secondary importance in view of the great fact that, prior to the introduction of iridectomy as a mode of treatment, glaucoma had always terminated in total blindness; while, after its introduction, a large number of cases were radically cured. It was soon ascertained by experience that the best results were obtainable in the acute forms, if taken in time, and that, in the chronic forms, little more was to be hoped for than an arrest of the malady at the point which it had reached. In other words, a retina which was suddenly and greatly compressed would be quickly destroyed, but was capable of complete recovery if the pressure were relieved with sufficient promptitude; while one which was compressed slowly and gradually sustained a smaller immediate deprivation of function, but, on the other hand, retained a smaller amount of recuperative power. Within narrow limits of time, a patient with acute glaucoma could be cured; and, within more extended limits of time, a patient with chronic glaucoma could be prevented from becoming blind.

In due course the effects of iridectomy in the hands of Von Graefe were made known in England, and the operation was employed at the Royal London Ophthalmic Hospital in all cases which appeared to be suitable for it, and chiefly by Mr. (afterwards Sir William) Bowman, by the late Mr. Critchett, and by Mr. Hulke. The senior surgeon, Mr. Dixon, long held himself aloof, but was ultimately convinced by the experience of his colleagues. The operation came to be regarded not only as legitimate, but as necessary, and Mr. Bowman took a leading part in advocating as well as in performing it. A paper which he read in 1863 before the annual meeting of the British Medical Association was intended to remove the whole question from the comparatively narrow domain of specialism, and to urge upon all medical practitioners the duty of rendering themselves acquainted with the symptoms of the early stages of glaucoma, and with the only method by which the progress of the disease could be arrested.

